# Outcomes After Initial Non-Operative Treatment of Osteochondral Lesions of the Talus (OLT) in Skeletally Immature Patients: A Cross-Sectional Study

**DOI:** 10.1177/19476035251357214

**Published:** 2025-07-23

**Authors:** Jason A.H. Steman, Tristan M.F. Buck, Jari Dahmen, Peter A.A. Struijs, Sjoerd A.S. Stufkens, Gino M.M.J. Kerkhoffs

**Affiliations:** 1 Department of Orthopedic Surgery and Sports Medicine, Amsterdam UMC, University of Amsterdam, Amsterdam, The Netherlands; 2 Musculoskeletal Health, Amsterdam Movement Sciences, Amsterdam, The Netherlands; 3 Academic Center for Evidence-based Sports medicine (ACES), Amsterdam, The Netherlands; 4 IOC Research Center, Amsterdam Collaboration on Health and Safety in Sports (ACHSS), Amsterdam, The Netherlands

**Keywords:** osteochondral lesions of the talus, skeletally immature, non-operative, ankle, cartilage

## Abstract

**Introduction:**

Literature on treatment outcomes in skeletally immature patients with osteochondral lesions of the talus (OLT) is scarce. As the healing of an OLT may be fundamentally different in a skeletally immature patient, more evidence is required focusing on this specific patient group. The primary aim of this study is to assess the conversion to surgery rate after initial non-operative management in skeletally immature patients with an OLT. The secondary aims of the present study are to assess and compare the clinical outcomes and reoperations after both non-operative and surgical treatment strategies at a mid- to long-term follow-up.

**Methods:**

All skeletally immature patients at the moment of initial treatment, treated for their primary or non-primary OLT with a minimum follow-up duration of 2 years, were included in this study. Patients with concomitant injuries were excluded. All patients started with non-operative management. In case of failure of non-operative management, patients converted to Bone Marrow Stimulation (BMS) or fixation. The primary outcome was the conversion to surgery rate after initial non-operative management. Secondary outcomes consist of reoperations at mature and immature age, pain during weight bearing, measured by the numeric rating scale (NRS), NRS of pain during rest, NRS during stair climbing, Berndt and Harty outcome question, Foot and Ankle Outcome Score (FAOS) and Short Form-36 (SF-36) and the patient satisfaction rate regarding the received treatment.

**Results:**

A total of 52 patients, 54% female, mean age of 13.6 years, were included in this study. Median follow-up duration was 81 months (range = 24-265 months). Seventeen patients received non-operative treatment as final treatment. In total, 35 (67%) out of 52 patients required surgical treatment after initial non-operative management, of which 14 underwent BMS and 20 had fixation while skeletally immature, 1 patient that had surgical treatment as an adult was excluded for further analysis. The median NRS of pain during weight bearing was 1 (interquartile range [IQR] = 0-2), 1 (IQR = 0-3), and 0 (IQR = 0-0.5) in the (sustained) non-operative, BMS, and fixation groups, respectively (*P* < 0.012). No significant differences in clinical outcomes between the different treatment groups could be observed. No complications occurred after surgical treatment. Reoperation rates were 21% and 20% in the BMS and fixation groups, respectively.

**Conclusions:**

The most important finding of this study is that 67% of the patients receiving initial non-operative management for OLTs ultimately required surgery.

**Level of evidence:**

Level III, cross-sectional comparative study

## Introduction

There is consensus that non-operative treatment is the first treatment in line in a standardized approach for the treatment of skeletally immature patients with an osteochondral lesion of the talus (OLT) as it can avoid surgery in a part of the patients.^1^ This treatment can consist of physical therapy, casting, avoidance of peak force, weight loss, or hyaluronic acid injections.^2,3^ In the pediatric population, non-operative treatment is indicated for all types of lesions, except for acute fixable lesions. The type of non-operative treatment depends on personal characteristics. Recently, the first prospective study on non-operative treatment for OLTs was published, showing a clinically relevant improvement in 38% of patients.^4^ However, this study included only adult patients, whose outcomes may differ from skeletally immature patients. A systematic review by Dahmen *et al.*^5^ specifically focusing on the skeletally immature population found that non-operative treatment was clinically successful in 4 out of 10 patients and, in general, surgical treatment was clinically successful in 7 out of 10 patients.

Despite the insights provided by this comprehensive review, it also highlighted the lack of high-quality, adequately powered studies that delve into treatment outcomes for skeletally immature OLT patients. This knowledge gap is a lamentable deficiency in the current state of research. Furthermore, the existing literature on pediatric OLTs falls short in elucidating treatment outcomes across the entire spectrum of the stepwise therapeutic approach, spanning from the initial non-operative management to the more invasive surgical strategies such as Bone Marrow Stimulation (BMS) and fixation. This shortcoming was also highlighted in the recently published consensus statement by Hurley *et al.*^6^ which underscored that there is a clear lack of high-quality clinical studies in the field of treatment of pediatric osteochondral lesions of the ankle. Consequently, this study aims to address these voids in the scientific and clinical knowledge along the entire treatment pathway, from non-operative management at the beginning to different types of surgical treatment at the end.

It is therefore the primary aim to study the conversion to surgery rate after initial non-operative management. The secondary aims consist of assessing the clinical outcomes after (sustained) non-operative and surgical management. We hypothesized that non-operative as well as surgical treatment may lead to satisfactory outcomes in the mid- to long-term.

## Methods

### Ethical Approval

This study is a non-randomized, non-blinded, cross-sectional comparative cohort study. Patients included in this study were asked for permission to review their electronic patient files and to fill out questionnaires. Medical ethical approval was obtained from the medical ethical committee of the Amsterdam University Medical Centers (Amsterdam UMC, W14_237#14.17.0288).

### Patient Selection

All skeletally immature patients (computed tomography [CT]-confirmed) treated for their OLT at the moment of initial treatment at our clinic were identified from the Amsterdam Ankle Cartilage Database. All identified patients having received their initial treatment between January 1990 and March 2021, that were actively followed up since their initial treatment, with a minimum of 2-year follow-up were invited to participate in this study. This invitation process consisted of a letter requesting permission and informed consent and used the information from the electronic patient file. Patients who fulfilled the below-described inclusion criteria and had fully completed the questionnaires were included. An overview of the inclusion and exclusion criteria is shown in Table 1.

**Table 1.** Inclusion and Exclusion Criteria.

Inclusion criteria	Exclusion criteria
Patients who received treatment for their OLT and were skeletally immature at the start of treatment, assessed using CT scans	Inability to fill out or not completely fulfilled questionnaires
Minimal 2-year follow-up	No informed consent given
Primary and non-primary OLTs	Patients with concomitant injuries
Both acute and chronic lesions	

### Data Collection

Data on baseline characteristics were acquired through reviewing the electronic patient files. The following data were extracted: age, gender, current follow-up duration, history of trauma, lesion location (lateral/medial), staging of the lesion, according to the modified Berndt and Harty classification system,^7^ and primary or secondary nature of the lesion. The primary nature of the lesion is defined as a lesion which is not surgical treated before. Secondary lesions are lesions that persist or develop after failed surgical management.

In addition, lesion size was measured in cranial-caudal (CC) diameter, medial-lateral (ML) diameter, anterior-posterior (AP) diameter, surface, and volume. Surface and volume were measured according to the ellipsoid formula.^8^ Furthermore, lesion morphology was described.^9^

Data regarding the type of treatment were obtained. Treatment groups included in this study consisted of fixation, BMS, and non-operative management. Furthermore, information on conversion to surgery and/or reoperations was obtained. All clinical outcomes in this study were obtained using the electronic data capture system CASTOR.

### Treatment Types

#### Non-operative management

All patients start with a non-operative treatment protocol for at least 6 months. Non-operative treatment aims to utilize the natural healing potential of the damaged tissue and to reduce the pain and inflammation within the joint. For acute non-displaced lesions as well as chronic lesions (minimum 6 months duration of symptoms), a non-operative treatment protocol is the first step in the treatment algorithm for OLTs.^3,10^ In our hospital, patients are treated using one of 3 possible non-operative treatment protocols. The first one being skillful neglect. Second, a cast-immobilization protocol which consists of a 4-week non–weightbearing cast after which a 4-week weightbearing cast is prescribed. Hereafter, patients will be prescribed with a walker for 4 weeks. During (and also after) this last period, patients will receive intensive and supervised therapy. The last option, only used for chronic lesions when a lack of strength and/or propriocepsis is present, is a supervised physiotherapy training with specific focus on strength, balance, intrinsic foot musculature, and propriocepsis, aiming at a gradual return to activity, potentially with the addition of inlays to correct for sinus tarsi pain and/or malalignment.^11^

Besides this standard protocol, other non-operative strategies that can be used consist of avoidance of peak force, which means that children cannot perform high-impact sports and should avoid activities like jumping, hyaluronic acid injections, or a skillful neglect.

#### Conversion to surgery

In case of failure of the above-described non-operative management, patients will convert to a surgical treatment, which can consist of BMS or fixation. Failure is defined as persistent symptoms after non-operative management that can be no further improved with non-operative management and is expected to improve with surgical treatment based on patient and radiological characteristics. The decision to conduct BMS or fixation depended on radiographic characteristic preoperatively.^3^ When a patient had a conversion to surgery at an immature age, the initial non-operative treatment was regarded as failed, and mid- to long-term clinical outcomes were obtained for the secondary treatment (i.e. BMS or fixation).

#### Bone marrow stimulation

Bone marrow stimulation is often the preferred surgical treatment option after failed non-operative treatment and is indicated especially for smaller osteochondral lesions.^2,3,12^ By removing all necrotic bone and overlying cartilage and drilling into the subchondral bone, BMS aims at the formation of a blood clot and the release of growth factors in order to form fibrocartilaginous tissue.

#### Fixation

The fixation technique is specifically indicated for lesions that have a fragmentous morphology.^13,14,15^ This technique can be conducted arthroscopically and open. By fixating the loose fragment to the underlying bone, this technique aims to reach union of the fragment in order to restore the natural congruency of the joint.

#### Reoperation

In this study, a reoperation was defined as a secondary surgical treatment aimed at the OLT itself (i.e. redo BMS after primary BMS or BMS after fixation), not including those aimed at resolving symptoms from any secondary causes (e.g. impingement or hardware removal). Patients who reached skeletal maturity at the moment of follow-up and had reoperation were included in this comparative analysis between patients who had, and who had no reoperation. As some included patients received multiple treatments while skeletally immature, the combined number of patients for all groups exceeds the total number of included patients. Clinical outcome measures were only collected for the last received treatment. For example, when a patient had a reoperation at an immature age, the initial treatment was regarded as failed, and mid- to long-term clinical outcomes were obtained for the secondary treatment. When a patient had a reoperation at a skeletally mature age, the treatment while skeletally immature was regarded as failed and no mid- to long-term clinical outcomes could be obtained through these questionnaires. These patients were therefore not analyzed through these questionnaires. However, these patients were included to calculate the reoperation/conversion to surgery rate and baseline characteristics of these patients were extracted, in order to compare patients that did convert to surgery or have a reoperation and those that did not.

### Outcome Measures

The primary outcome is the rate of patients who converted to surgery after initial non-operative management. Secondary clinical outcome measures were the Numeric Rating Scale (NRS) of pain during weight bearing, the NRS of pain during rest, the NRS of during stair climbing and NRS running, Foot and Ankle Outcome Score (FAOS), Short Form-36 (SF-36) after sustained non-operative treatment, BMS, or fixation. Number and type of complications after surgical treatment were reported, as well as the reoperation rates after BMS and fixation. In addition, all clinical outcomes were compared between the different treatment strategies.

The NRS is a subjective pain scale ranging from 0, meaning no pain, to 10, meaning the worst pain imaginable.^16^ The FAOS is a patient-reported outcome measure, which consists of 42 questions divided between 5 subscales: symptoms, pain, activities of daily living, sport, and quality of life.^17^ The SF-36 is an outcome measure that has been extensively used to assess quality of life. It consists of a mental and physical components scale, with scores ranging from 0 to 100 and a higher score indicating a better health status.

### Statistical Analysis

Baseline characteristics were reported in mean and standard deviation in the case of normally distributed data. In case of non-normality, median and interquartile range (IQR) were used. A Shapiro-Wilk test was used to assess data for normality, as well as a visual inspection using boxplots. For categorical variables, absolute numbers and percentages were used. For comparison of baseline characteristics and outcome measures between the reported groups (non-operative, BMS, fixation) either 1-way analysis of variance (ANOVA) or the Kruskal-Wallis test was used for numerical data depending on the normality of the data. A chi-square test was used for the comparison of categorical data. For numerical data, in case of significant differences, the Wilcoxon rank sum test was used to compare the outcomes between individual groups. For the comparison of numeric variables between the patients who had no conversion/reoperation and the patients who had conversion/reoperation, the Mann-Whitney *U*-test or the non-parametric unpaired *T*-test was used depending on the normality of the data. For comparison of categorical variables in this comparison, the chi-squared test was used.

For the comparison between the 3 groups (non-operative, BMS, lift, drill, fill, and fix [LDFF]), a significance level of 0.012 was used in order to correct for multiple testing. For the comparison between the conversion/reoperation group and no conversion/reoperation group, a significance level of 0.05 was used. The statistical analyses were performed using SPSS software v28.

For all radiological characteristics, an intraclass correlation coefficient (ICC) value was calculated in order to improve test the reliability of the measurement. A 2-way random ICC model was used to calculate the inter-observer agreement. The ICC was interpreted as poor (0.40); moderate (0.40-0.75); substantial (0.75-0.90); or excellent reliability (>0.90).^18^

## Results

### Study Population

**Figure 1.**
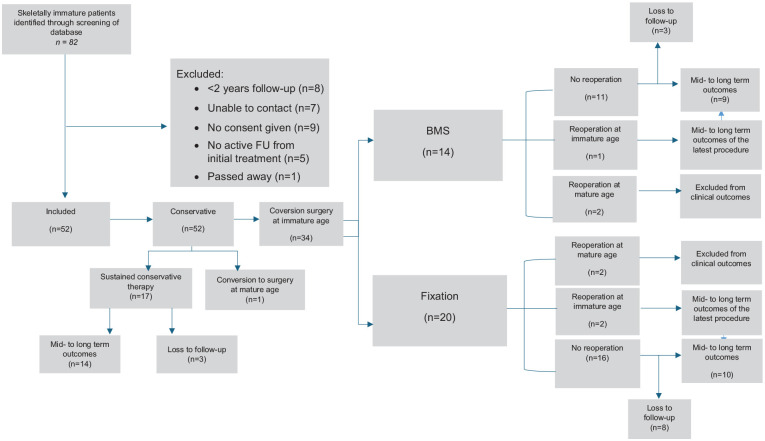
Flowchart inclusion process. BMS = bone marrow stimulation; FU = follow-up.

A total of 82 skeletally immature patients were screened for eligibility, of which 52 patients were included. The overview of the selection process, including reasons for exclusion, is presented in Figure 1. The mean age at the time of treatment was 13.6 years (SD ±1.8 years), and the percentage of females and males was 54% and 46%, respectively. Median follow-up duration was 81 months (range = 24-265 months). In total, 48 patients had primary lesion (92%) and 4 secondary lesion (8%). The mean lesion size was AP 12.9 mm (SD ±4.6 mm), ML 9.6 mm (SD ±2.1 mm), and depth 6.3 mm (SD ±2.7 mm). An inter-observer reliability of 0.94 was obtained for the radiological measures, which is considered as excellent. In total, 26 of 52 patients reported a history of trauma (50%). An overview of the patient selection and allocation of treatment is shown in Figure 1.

### Treatment Groups

#### Non-operative management

All patients initially started with non-operative management, after which 34 out of the 52 identified patients (65%) converted to surgical treatment while skeletally immature. This left a total of 18 patients to be included within the non-operatively treated group. Of these 18 patients, 1 patient (7%) eventually converted to surgery at a skeletally mature age and was therefore not included in the analysis of the mid- to long-term follow-up clinical outcomes.

This results in a total of 17 patients who received sustained non-operative management, of which 3 were lost to follow-up. For these 3 patients, only baseline characteristics could be obtained and whether they converted to surgery. In this group of 17 patients, the following non-operative modalities were applied: (1) physical therapy was applied for 7 patients, (2) a casting protocol was used for 2 patients, (3) skillful neglect and avoidance of peak force were applied for 7 patients, and (4) hyaluronic acid injections were used for 1 patient. In total, clinical outcomes could be obtained for 14 patients. Clinical outcomes are presented in Table 2.

**Table 2.** Clinical Outcomes of the Sustained Non-operative Treatment Group.

Pain Scores (NRS), median (IQR)	
NRS during weight bearing	1.0 (0-2.0)
NRS in rest	0.5 (0-1.0)
NRS during running	3.0 (1.0-4.0)
NRS during stair climbing	1.0 (0-1.5)
FAOS scores, median (IQR)	
Pain	94 (83.33-100)
QOL	56.2 (43.75-67.2)
Sport	75 (62.5-90)
Symptoms	82.14 (71-91.1)
ADL	100 (100-100)
SF-36, median (IQR)	
PCS	49.9 (46.8-51.6)
MCS	50.9 (48.3-53.2)
Satisfaction, n (%)	
Satisfied	12 (86%)
Neutral	2 (22%)

n = number; n.s. = not significant; SD = standard deviation; IQR = interquartile range.

#### Conversion versus no conversion to surgery

A comparison of baseline patient and lesion characteristics between the group of patients that required surgical intervention and the group that did not require surgical intervention showed that both fragmentous and lateral lesions were seen more often in the group that did require surgery. An overview and comparison of baseline patient and lesion characteristics for these groups are shown in Table 3.

**Table 3.** Overview and Comparison of Patient- and Lesion Characteristics for Non-operative and Surgically Treated Groups.

	No Conversion to Surgery (*n* = 18)	Conversion to Surgery (*n* = 34)	*P*
Sex, *n* (% male)	10 (56%)	14 (41%)	n.s.
Age at treatment (years), mean ± SD	13.1 ± 2.0	13.9 ± 1.6	n.s.
Previous ankle trauma, *n* (%)	9 (50%)	17 (50%)	n.s.
Lesion size (mm), mean ± SD			
Anterior-posterior	13.0 ± 4.1	14.4 ± 4.3	n.s.
Medial-lateral	8.9 ± 1.7	8.9 ± 2.2	n.s.
Depth	5.5 ± 2.4	6.0 ± 2.3	n.s.
Lesion location, *n* (%)			
Medial	17 (94%)	21 (62%)	0.007
Lateral	1 (6%)	13 (38%)	
Lesion aspect, *n* (%)			
Fragmentary	6 (33%)	26 (81%)	0.002
Crater	12 (66%)	8 (19%)	
Cystic	0 (0%)	0 (0%)	
Berndt and Harty staging, *n* (%)	1 stage I (6%)	0 stage I (0%)	n.s.
	9 stage II (50%)	7 stage II (21%)	
	6 stage III (33%)	16 stage III (47%)	
	0 stage IV (0%)	2 stage IV (6%)	
	2 stage V (11%)	9 stage V (26%)	

n = number; n.s. = not significant; SD = standard deviation.

### Surgical Treated Groups

#### Bone marrow stimulation

Bone marrow stimulation as the first surgical strategy was applied in 14 identified patients within this group, of which 11 were primary and 3 were secondary BMS. Of these 14 patients, 3 were lost to follow-up, and for this group only baseline characteristics and information on complications and whether they had a reoperation could be collected. No complications occurred after treatment with BMS. In total, 3 patients (21%) had a reoperation, 2 patients had a redo of their BMS at mature age, and 1 patient had a redo BMS at immature age. The median time to reoperation was 22 months (range = 14-30). The patients that had a reoperation at a mature age were not included in the analysis of the mid- to long-term follow-up clinical outcomes. This results in a total of 9 patients for whom clinical outcomes were obtained. Clinical outcomes are presented in Table 4.

**Table 4.** Patient and Lesion Characteristics of the Bone Marrow Stimulation Group.

	BMS (*n* = 9)
Sex, *n* (% male)	2 (22%)
Age at treatment (years), mean ± SD	13.9 ± 1.1
BMI (kg/m^2^), mean ± SD	26.1 ± 3.3
Primary/secondary defect, *n* (% secondary)	2 (22%)
Previous ankle trauma, *n* (%)	5 (56%)
Follow-up duration (months), mean ± SD	125.2 ± 89.7
Lesion laterality, *n* (%)	
Left	3 (33%)
Right	6 (66%)
Bilateral	0 (0%)
Lesion location, *n* (%)	
Medial	7 (78%)
Lateral	2 (22%)
Lesion aspect, *n* (%)	
Fragmentary	1 (11%)
Crater	8 (89%)
Cystic	0 (0%)
Lesion size (mm), mean ± SD	
Anterior-posterior	14.3 ± 2.9
Medial-lateral	9.5 ± 3.2
Depth	5.6 ± 2.5
Berndt and Harty, *n* (%)	0 stage I (0%)
	3 stage II (33%)
	2 stage III (22%)
	0 stage IV (0%)
	4 stage V (44%)

n = number; n.s. = not significant; SD = standard deviation; IQR = interquartile range.

#### Fixation

Fixation as first surgical treatment was applied in 20 patients, for 21 ankles, of which 10 were treated by open fixation and 11 were treated through arthroscopic fixation. No complications occurred after treatment with fixation. Of the 20 patients within this group, 4 patients (20%) had a reoperation with a median time to reoperation of 10 months (range = 6-20). Two patients had a reoperation at a mature age, and 2 patients had a reoperation at an immature age. In the group of patients who had a reoperation at an immature age, both patients had a refixation. Patients who had a reoperation at mature age or other than fixation were not included in the analysis of the mid- to long-term follow-up clinical outcomes. In total, 8 patients were lost to follow-up, and for this group only information on baseline characteristics and whether they had a reoperation could be obtained. This results in a total of 10 patients for which clinical outcomes were collected. Clinical outcomes are presented in Table 5.

**Table 5.** Patient and Lesion Characteristics of the Fixation Group.

	Fixation (*n* = 10)
Sex, *n* (% male)	9 (90%)
Age at treatment (years), mean ± SD	14.3 ± 1.4
BMI (kg/m^2^), mean ± SD	26.4 ± 5.8
Primary/secondary defect, *n* (% secondary)	2 (20%)
Previous ankle trauma, *n* (%)	5 (50%)
Follow-up duration (months), mean ± SD	83.6 ± 61.9
Lesion laterality, *n* (%)	
Left	6 (60%)
Right	3 (30%)
Bilateral	1 (10%)
Lesion location, *n* (%)	
Medial	4 (40%)
Lateral	6 (60%)
Lesion morphology	
Fragmentary	10 (100%)
Crater	0 (0%)
Cystic	0 (0%)
Lesion size (mm), mean ± SD	
Anterior-posterior	14.4 ± 5.1
Medial-lateral	8.9 ± 2.4
Depth	6.5 ± 2.5
Berndt and Harty, *n* (%)	0 stage I (0%)
	1 stage II (10%)
	6 stage III (60%)
	2 stage IV (20%)
	1 stage V (10%)

n = number; n.s. = not significant; SD = standard deviation; IQR = interquartile range.

### Comparison

No significant differences in clinical outcomes were observed between the different treatment groups. An overview and comparison of outcomes per treatment group are shown in Table 6.

**Table 6.** Overview and Comparison of Clinical Outcomes Per Treatment Group.

	Non-operative (*n* = 14)	BMS (*n* = 9)	Fixation (*n* = 10)	*P*
Pain scores (NRS), median (IQR)				
NRS during weight bearing	1.0 (0-2.0)	1.0 (0-3.0)	0 (0-0.5)	n.s.
NRS in rest	0.5 (0-1.0)	0 (0-1.5)	0 (0-0)	n.s.
NRS during running	3.0 (1.0-4.0)	3.5 (0.5-6.5)	1.0 (0-2)	n.s.
NRS during stair climbing	1.0 (0-1.5)	0.5 (0-3.5)	0 (0-0)	n.s.
FAOS scores, median (IQR)				
Pain	94 (83.33-100)	91.8 (69.4-99.3)	97.2 (81.9-100)	n.s.
QOL	56.2 (43.75-67.2)	53.1 (31.2-89.2)	75 (56.3-93.8)	n.s.
Sports	75 (62.5-90)	82.5 (36.3-92.5)	75 (58.5-92.5)	n.s.
Symptoms	82.14 (71-91.1)	71.4 (57.1-85)	78.6 (67.9-84)	n.s.
ADL	100 (100-100)	100 (98.4-100)	100 (100-100)	n.s.
SF-36, median (IQR)				
PCS	46.8 (37.6-49.9)	48.4 (39.0-50.8)	52.7 (49.3-53.7)	n.s.
MCS	45.1 (41.7-50.9)	55.9 (43.3-59.3)	54.8 (47.8-58.2)	n.s.
Satisfaction, n (%)				
Satisfied	12 (86%)	6 (66%)	9 (90%)	n.s.
Neutral	2 (22%)	2 (22%)	1 (10%)	n.s.
Unsatisfied	0 (0%)	1 (11%)	0 (0%)	n.s.
Would undergo treatment again, *n* (%)	12 (86%)	7 (78%)	10 (100%)	n.s.

n = number; IQR = interquartile range; NRS = numeric rating scale; QOL = quality of life; ADL = activities of daily living; PCS = physical components scale; MCS = mental components scale; BMS = bone marrow stimulation; n.s. = not significant.

## Discussion

The most important finding of this study is that 67% of the patients receiving initial non-operative management for OLTs ultimately required surgery, including 1 patient at a later stage, while skeletally mature. In addition, (sustained) non-operative treatment, as well as BMS and fixation after failure of non-operative treatment, showed good mid- to long-term clinical results for treatment of skeletally immature patients.

This is the first study describing outcomes during the whole stepwise treatment pathway from initial non-operative treatment to different types of surgical treatment. As the existing literature on pediatric OLTs falls short in elucidating treatment outcomes across the entire spectrum of the stepwise therapeutic approach, this study added value to the current literature as it described outcomes along the whole pathway. The results of this study aid physicians, patients, and their caretakers in the treatment process and its corresponding outcomes for all stages of the treatment process. In addition, it will help to manage the expectations of patients and their caretakers.

### Clinical Outcomes

A conversion to surgery rate (either while still skeletally immature or at a later age) after initial non-operative treatment of 65% was found, leaving 35% of patients being successfully treated using a non-operative protocol. In this case, successful was defined as no conversion to surgery, as this might be assumed as an acceptable level of functioning. This conversion to surgery rate is roughly the same as the rate of 62% found in the systematic review by Dahmen *et al.*^5^ The results of this study show, however, that the percentage of patients converting to surgery at a later age (when skeletally mature) was low (7%) and for the group of patients that initially did not convert to surgery, satisfying mid- to long-term clinical results were seen in this study. These findings support the consensus that all OLTs, except for acute stage IV lesions, should be initially managed using a non-operative treatment protocol.^12^ However, optimalization and personalization of the non-operative treatment strategy may further improve clinical outcomes. For example, weight loss should be indicated standardly in patients with an increased body mass index (BMI), as it is known that an increased BMI is associated with lesion size.^19^ For that reason, future studies need to focus on a standardized and personalized evidence-based approach in order to optimize non-operative treatment. In this study, a significantly higher rate of conversion to surgery was seen in lesions with a fragmentary morphology. A recent retrospective study on conversion to surgery in OLTs also showed a relatively high hazard ratio when a fragment was present, although not significant.^20^ This study also found a significant higher rate of conversion to surgery for lateral lesions. However, as this is in contrast with other reported outcomes and all these lateral lesions had a fragmentary morphology, we believe that this difference can be attributed to the lesion morphology rather than location.

Within this study, fixation was the most frequently used surgical treatment strategy. Satisfying mid- to long-term results were found, which is also reported in the current literature. Kumai *et al.*^21^ found good Berndt and Harty scores in 24 out of 27 patients (89%), of which 5 out of 5 (100%) skeletally immature patients. Furthermore, a case series by Dunlap *et al.*^22^ showed 6 good (60%), 3 fair (30%), and 1 poor outcome (10%). This series included 5 skeletally immature patients for whom 4 good (80%), 0 fair (0%), and 1 poor (20%) outcomes were found.

Treatment of OLTs using BMS is generally chosen for patients with relatively small lesions.^5^ In the present study, when looking at the primary outcome, satisfying results after treatment with BMS were seen. A median NRS of 1 during weight bearing was found, along with low pain scores in other categories as well. A known drawback of treatment with BMS is the formation of fibrocartilage tissue as opposed to the original hyaline cartilage.^3^ Due to the inferior wear characteristics of this fibrocartilage tissue, it could deteriorate over time and might cause progression of osteoarthritis (OA).^23^ Compared to the current literature, our findings were similar to the study of Reilingh *et al.*^2^ and D’Ambrosi *et al.*^24^ which showed good clinical outcomes in terms of the primary outcome, pain during weight bearing. However, it also showed that patients who had BMS were less satisfied with their result compared to fixation, which was similar in the outcomes of the current study.

Based on all above-mentioned outcomes of the current study and the reflection of these outcomes on the current literature, this study supports the treatment algorithm that starts with non-operative treatment, as it can avoid surgery in a certain number of patients.^1,5^ This non-operative treatment may consist of (a combination of) three different types: (1) supervised neglect, (2) supervised physiotherapy training with specific focus on strength, balance, intrinsic foot musculature, and (3) propriocepsis aiming at a gradual return to activity, potentially with the addition of inlays to correct for sinus tarsi pain and/or malalignment. Currently, no evidence exists regarding a superior non-operative strategy. Therefore, the type of non-operative treatment is decided through a shared decision-making process. In case non-operative treatment does not provide a sufficient improvement, surgical treatment strategies can be considered as the next step. In this study, BMS and fixation both provided satisfactory outcomes and are therefore both good options in case of failure of non-operative treatment. These treatment options showed no statistical difference in clinical outcomes in this study. However, as fixation restores the joint congruity and preserves the hyaline cartilage layer, this surgical treatment option is preferred when feasible, for fragmentary lesions with a minimum depth of 3 mm.^25^ For larger lesions, another alternative is to wait for the physes to close, so that a medial malleolar osteotomy can be performed for an osteo(chondral) transplantation procedure.

### Reoperations

Reoperations after BMS (21%) and fixation (20%) were relatively low compared to the conversion to surgery after initial non-operatively treated group. Despite the fact that direct surgical treatment may result more often in definitive resolution of complaints, non-operative treatment still may be the most suitable initial treatment, as it can avoid surgery in 33% of the patients. Based on the comparison between conversion to surgery after initial non-operative treatment, it can be stated that no influencing factors were identified that increases the risk of conversion to surgery, as none of the factors differs significantly between the patients who had and who had no conversion to surgery.

#### Strengths and limitations

This study has several strengths. It is the largest case series to date in the scientific literature on OLTs solely including skeletally immature patients during their initial treatment. The long follow-up duration adds to the existing knowledge, as few studies with such follow-up times exist within this specific patient group.

This study also has its limitations. The first limitation is the combination of the already small number of patients within each specific treatment group, which makes that the number of patients within each treatment group is insufficient to perform any meaningful statistical analysis. Furthermore, no radiological results were included in this study, which results in the lack of information about degenerative changes over time. In addition, no MRIs were used at baseline and follow-up which made it impossible to estimate the amount of bone marrow edema, which may influence the outcome of non-operative management.^26^ Another limitation is the time span of 27 years in outcomes used in this study. Due to this long time, non-operative strategies may be changed, which causes heterogeneity in the used non-operative strategies. This heterogeneity may influence outcomes of non-operative treatment. Furthermore, the high number of patients was lost to follow-up. Due to the cross-sectional design of the study, with a long follow-up period, we were unable to include several patients, as their contact details had changed and we were unable to reach them. Moreover, some patients did not want to participate, likely since they received their treatment several years ago. However, it is unlikely that this might influence the results, as there are still a large number of included patients. Finally, the current study does not provide any sports-related results. As OLTs are often seen in the athletic population, these results might be an important factor in the decision-making process.

## Conclusions

The most important finding of this study is that 67% of the patients receiving initial non-operative management for OLTs ultimately required surgery. In addition, (sustained) non-operative treatment, as well as BMS and fixation after failed non-operative management, showed good mid- to long-term clinical results for treatment of skeletally immature patients, with a median NRS of pain during weight bearing of 1, 1, and 0. No significant differences in outcomes between these treatment options could be observed.

## References

[1] Dombrowski ME Yasui Y Murawski CD Fortier LA Giza E Haleem AM , et al Conservative management and biological treatment strategies: proceedings of the international consensus meeting on cartilage repair of the ankle. Foot Ankle Int. 2018;39(1 Suppl):9S-15S. 10.1177/1071100718779390 30215314

[2] Reilingh ML Kerkhoffs GM Telkamp CJ Struijs PA van Dijk CN. Treatment of osteochondral defects of the talus in children. Knee Surg Sports Traumatol Arthrosc. 2014;22(9):2243-9. doi:10.1007/s00167-013-2685-7. 24045918

[3] Rikken QGH Kerkhoffs GMMJ. Osteochondral lesions of the talus: an individualized treatment paradigm from the Amsterdam perspective. Foot Ankle Clin. 2021;26(1):121-36. doi:10.1016/j.fcl.2020.10.002. 33487235

[4] Buck TMF Steman JAH Dahmen J Rikken QGH Sierevelt IN Stufkens SAS , et al Nonoperative treatment for osteochondral lesions of the talus provides clinical improvement in the minority of the patients at short-term follow-up. Foot Ankle Int. Epub 2025 Apr 22. doi:10.1177/10711007251330881. PMC12227802 40261033

[5] Dahmen J Steman JAH Buck TMF Struijs PAA Stufkens SAS van Bergen CJA , et al Treatment of osteochondral lesions of the talus in the skeletally immature population: a systematic review. J Pediatr Orthop. 2022;42(8):e852-e860. doi:10.1097/bpo.0000000000002175. PMC9351694 35605211

[6] Hurley DJ Davey MS Hurley ET Murawski CD Calder JDF D’Hooghe P et al. Paediatric ankle cartilage lesions: proceedings of the international consensus meeting on cartilage repair of the ankle. J ISAKOS. 2022;7(5):90-4. doi:10.1016/j.jisako.2022.04.001. 35774008

[7] Loomer R Fisher C Lloyd-Smith R Sisler J Cooney T. Osteochondral lesions of the talus. Am J Sports Med. 1993;21(1):13-9. doi:10.1177/036354659302100103. 8427354

[8] Choi WJ Park KK Kim BS Lee JW. Osteochondral lesion of the talus: is there a critical defect size for poor outcome? Am J Sports Med. 2009;37(10):1974-80. doi:10.1177/0363546509335765. 19654429

[9] van Diepen PR Smithuis FF Hollander JJ Dahmen J Emanuel KS Stufkens SAS , et al Reporting of morphology, location, and size in the treatment of osteochondral lesions of the talus in 11,785 patients: a systematic review and meta-analysis. Cartilage. Epub 2024 Feb 16. doi:10.1177/19476035241229026. PMC11569679 38366391

[10] Murawski CD Jamal MS Hurley ET Buda R Hunt K McCollum G et al. Terminology for osteochondral lesions of the ankle: proceedings of the international consensus meeting on cartilage repair of the ankle. J ISAKOS. 2022;7(2):62-6. doi:10.1016/j.jisako.2021.12.001. 35546437

[11] Buck TMF Lauf K Dahmen J Altink JN Stufkens SAS Kerkhoffs GMMJ . Non-operative management for osteochondral lesions of the talus: a systematic review of treatment modalities, clinical- and radiological outcomes. Knee Surg Sports Traumatol Arthrosc. 2023;31(8):3517-27. doi:10.1007/s00167-023-07408-w. PMC10356662 37062042

[12] 2019 International Consensus Group on Cartilage Repair of the Ankle. Pediatric ankle cartilage lesions: proceedings of the international consensus meeting on cartilage repair of the ankle. J ISAKOS. 2022;7:90-4. 10.1016/j.jisako.2022.04.001 35774008

[13] Reilingh ML Lambers KTA Dahmen J Opdam KTM Kerkhoffs GMMJ . The subchondral bone healing after fixation of an osteochondral talar defect is superior in comparison with microfracture. Knee Surg Sports Traumatol Arthrosc. 2018;26(7):2177-82. doi:10.1007/s00167-017-4654-z. PMC6061443 28752185

[14] Kerkhoffs GM Reilingh ML Gerards RM de Leeuw PA. Lift, drill, fill and fix (LDFF): a new arthroscopic treatment for talar osteochondral defects. Knee Surg Sports Traumatol Arthrosc. 2016;24(4):1265-71. doi:10.1007/s00167-014-3057-7. 24841940

[15] Rikken Q Favier B Dahmen J Stufkens S Kerkhoffs G. Open lift–drill–fill–fix for medial osteochondral lesions of the talus: surgical technique. Offene lift-drill-fill-fix-methode bei medialen osteochondralen läsionen des talus: operationstechnik. Operative Orthopädie Und Traumatologie. 2024;36:132-44. doi:10.1007/s00064-023-00833-7. PMC11014820 37828133

[16] Gagliese L Weizblit N Ellis W Chan VWS . The measurement of postoperative pain: a comparison of intensity scales in younger and older surgical patients. Pain. 2005;117(3):412-20. doi:10.1016/j.pain.2005.07.004. 16153776

[17] Sierevelt IN Beimers L van Bergen CJA Haverkamp D Terwee CB Kerkhoffs GMMJ . Validation of the Dutch language version of the foot and ankle outcome score. Knee Surg Sports Traumatol Arthrosc. 2015;23(8):2413-9. doi:10.1007/s00167-014-3017-2. 24792074

[18] Terwee CB Bot SD de Boer MR van der Windt DA Knol DL Dekker J et al. Quality criteria were proposed for measurement properties of health status questionnaires. J Clin Epidemiol. 2007;60(1):34-42. doi:10.1016/j.jclinepi.2006.03.012. 17161752

[19] D’Ambrosi R Maccario C Serra N Ursino C Usuelli FG. Relationship between symptomatic osteochondral lesions of the talus and quality of life, body mass index, age, size and anatomic location. Foot Ankle Surg. 2018;24(4):365-72. doi:10.1016/j.fas.2017.04.011. 29409207

[20] Buck TMF Dahmen J Altink JN Rikken QGH Sierevelt IN Stufkens SAS , et al Higher age is associated with lower likelihood of conversion to surgery after primary nonoperative treatment for osteochondral lesions of the talus. Cartilage. Epub 2024 Jan 26. doi:10.1177/19476035241227357. PMC11569626 38279550

[21] Kumai T Takakura Y Kitada C Tanaka Y Hayashi K. Fixation of osteochondral lesions of the talus using cortical bone pegs. J Bone Joint Surg Br. 2002;84(3):369-74. doi:10.1302/0301-620x.84b3.12373. 12002495

[22] Dunlap BJ Ferkel RD Applegate GR. The “LIFT” lesion: lateral inverted osteochondral fracture of the talus. Arthroscopy. 2013;29(11):1826-33. doi:10.1016/j.arthro.2013.08.012. 24209680

[23] van Bergen CJA Kox LS Maas M Sierevelt IN Kerkhoffs GMMJ van Dijk CN. Arthroscopic treatment of osteochondral defects of the talus: outcomes at eight to twenty years of follow-up. J Bone Joint Surg Am. 2013;95(6):519-25. doi:10.2106/jbjs.L.00675. 23515986

[24] D’Ambrosi R Maccario C Ursino C Serra N Usuelli FG. Combining microfractures, autologous bone graft, and autologous matrix-induced chondrogenesis for the treatment of juvenile osteochondral talar lesions. Foot Ankle Int. 2017;38(5):485-95. 10.1177/1071100716687367 28076977

[25] Reilingh ML Murawski CD DiGiovanni CW Dahmen J Ferrao PNF Lambers KTA , et al Fixation techniques: proceedings of the international consensus meeting on cartilage repair of the ankle. Foot Ankle Int. 2018;39(1 Suppl):23S-27S. doi:10.1177/1071100718781096. 30215310

[26] D’Ambrosi R Maccario C Ursino C Serra N Usuelli FG. The role of bone marrow edema on osteochondral lesions of the talus. Foot Ankle Surg. 2018;24:229-35. 10.1016/j.fas.2017.02.010 29409254

